# Therapeutic Switching of Biological Drugs in Paediatric Severe Asthma: Experience of a Referral Centre

**DOI:** 10.3390/biomedicines14092050

**Published:** 2026-09-11

**Authors:** Valentina Agnese Ferraro, Margherita Amadi, Veronica Ferasin, Stefania Zanconato, Silvia Carraro

**Affiliations:** 1 Women’s and Children’s Health Department—SDB, University of Padua, 35128 Padua, Italyveronica.ferasin@aopd.veneto.it (V.F.); silvia.carraro@unipd.it (S.C.); 2 Unit of Pediatric Allergy and Respiratory Medicine, Women’s and Children’s Health Department, University Hospital of Padua, 35128 Padua, Italy; stefania.zanconato@aopd.veneto.it

**Keywords:** pediatric severe asthma, switching biologic therapy, real-world tertiary care setting

## Abstract

**Background/Objectives**: Biologic therapies are the cornerstone of severe asthma management. However, evidence regarding switching between biologics in children with severe asthma remains limited. **Methods**: Retrospective observational study including patients aged 6–17 years with severe asthma, followed at our tertiary referral center, who switched biologic therapy. Clinical, laboratory, and lung function data were collected at the initiation of the second biologic (T0) and after 4 (T1) and 12 months (T2). Changes in asthma control, exacerbation rate, inhaled corticosteroid dose, FeNO and lung function were evaluated. **Results**: Eight patients who switched and had been treated with a second biologic for at least 12 months were included. Switching occurred from omalizumab to dupilumab (*n* = 1), mepolizumab to dupilumab (*n* = 5), and mepolizumab to omalizumab (*n* = 2). The main reason for switching was inadequate asthma control, while one patient switched because of poorly controlled comorbidity. At T2, exacerbations significantly decreased (*p* = 0.03), with significant improvements in FEV_1_ (*p* = 0.002) and FEV_1_/FVC (*p* = 0.015). The daily fluticasone-equivalent dose decreased from a median of 500 μg to 425 μg. **Conclusions**: In this cohort, switching biologic therapy was associated with improved clinical and functional outcomes, with a significant response already detectable after 4 months. These findings highlight the importance of close clinical and functional monitoring to identify children who may benefit from a change in biologic therapy. Larger prospective studies are needed to define standardized criteria and optimal timing for biologic switching in pediatric severe asthma.

## 1. Introduction

Severe asthma is a heterogeneous chronic inflammatory disease that remains uncontrolled despite treatment with high-dose inhaled corticosteroids, additional controller medications, and optimal management of comorbidities [1]. It is associated with frequent exacerbations, accelerated decline in lung function, and impaired quality of life, and has a substantial healthcare burden [2]. Advances in the characterization of asthma phenotypes and endotypes have enabled the development of targeted biologic therapies, marking a paradigm shift toward personalized treatment strategies for patients with severe disease [3]. Key mediators of type 2 inflammation, including interleukin (IL)-5, IL-4, IL-13, and immunoglobulin E (IgE), represent the principal targets of currently available biologic therapies [4]. Four monoclonal antibodies are currently approved for pediatric use in Europe and Italy—omalizumab, mepolizumab, dupilumab, and tezepelumab—each targeting a distinct component of the inflammatory cascade [5]. In addition to reducing exacerbation rates and corticosteroid exposure, these therapies have raised the possibility of modifying the course of the disease when initiated during childhood [6]. The selection of biologic therapy for children with severe asthma should be guided by a comprehensive assessment of both the clinical phenotype and the underlying inflammatory endotype, integrating individual patient characteristics, comorbidities, and type 2 biomarkers within a precision medicine framework [7]. However, no head-to-head clinical trials have directly compared the efficacy of the biologics currently approved for pediatric use, and evidence-based recommendations for selecting a first-line agent in children eligible for multiple therapies are lacking [8]. Treatment response should therefore be reassessed regularly, as changes in disease activity, clinical characteristics, or biomarker profiles may support switching to an alternative biologic in the event of an inadequate response, adverse events, or evolving therapeutic indications [5,9,10]. Today, robust evidence and standardized guidelines regarding biologic switching in pediatric severe asthma remain limited.

This study aimed to characterize children with severe asthma who underwent biologic switching in a real-world tertiary care setting, identify the clinical factors that prompted treatment modification, and evaluate the effects of switching on asthma control, exacerbation frequency, lung function, and inhaled corticosteroid use over a 12-month follow-up.

## 2. Materials and Methods

### 2.1. Study Design and Ethical Approval

This retrospective observational study included children and adolescents (6–17 years) with severe asthma followed in the Pediatric Allergy and Respiratory Medicine Unit, Department of Women’s and Children’s Health, University Hospital of Padova (Italy). The study was approved by the local Ethics Committee (CET-ACEV 6195/AO/25; 10 April 2025), and written informed consent was obtained from parents or legal guardians, with assent from minors when appropriate.

### 2.2. Study Population and Data Collection

Eligible patients were those who underwent a switch from one biologic therapy to another during follow-up and continued the second biologic for at least 12 months. Clinical data were retrospectively collected from medical records at biologic switch (T0), 4 months (T1), and 12 months (T2) after initiation of the second biologic. Demographic characteristics, indications for switching, comorbidities, maintenance asthma treatment, exacerbation rate, GINA symptom control, Fractional exhaled nitric oxide (FeNO), lung function, and pediatric asthma-related quality of life (PAQLQ) were analyzed. Asthma exacerbation was defined as an episode requiring treatment with systemic corticosteroids for more than 3 consecutive days. Spirometry was performed using a bell spirometer (Biomedin, Padua, Italy). The spirometric parameters are expressed as a percentage of the predicted values and z-scores according to the reference equations of the Global Lung Function Initiative (GLI-2012) endorsed by the European Respiratory Society (ERS). Pulmonary function was assessed at the time of biologic switch (T0) and after 4 (T1) and 12 months (T2). FeNO was measured using a portable electrochemical analyzer (NIOX VERO, Aerocrine, Solna, Sweden).

As no validated and universally accepted definition of non-response to biologic therapy is currently available for children with severe asthma [11], the decision to switch was based on a multidimensional clinical assessment rather than on prespecified thresholds for individual outcomes. In accordance with our center’s clinical practice, patients were evaluated by a pediatric pulmonologist every 2 to 4 weeks—depending on biologic dosing schedule—for the first 6 months of treatment, and every 2 months thereafter. Patients were considered non-responders when, after at least 6 months of treatment, they showed limited or no clinically meaningful improvement, or experienced worsening despite treatment. Treatment response was assessed by considering exacerbation burden and need for oral corticosteroids, symptom control, lung function, treatment adherence and comorbidities. Accordingly, no isolated FEV_1_ threshold or predefined minimum number of exacerbations was used as the sole criterion for switching.

### 2.3. Statistical Analysis

Statistical analyses were performed using descriptive statistics, and continuous variables are presented as median and interquartile range (IQR). Comparisons across the three study time points (T0, T1, and T2) were performed using repeated-measures analysis of variance (ANOVA) for normally distributed variables (spirometric parameters) and the Friedman test for non-normally distributed variables (GINA symptom control score). Changes in the annual number of exacerbations and daily inhaled corticosteroid (ICS) dose between T0 and T2 were assessed using the Wilcoxon signed-rank test for paired data. A two-sided *p* value < 0.05 was considered statistically significant. Given the small sample size, all analyses should be considered exploratory, and the results should be interpreted cautiously because of the limited statistical power and potential instability of the estimates. All statistical analyses were performed using GraphPad Prism software (version 10.6.1; GraphPad Software, San Diego, CA, USA). The use of GenAI for superficial text editing (e.g., grammar, spelling, punctuation, and formatting) does not need to be declared.

## 3. Results

### 3.1. Clinical Characteristics

A total of 8 patients (5 males) were included in the study. Patients’ characteristics are described in Table 1.

The median age at initiation of the first biologic was 11.1 years (range, 7.5–12.6), whereas the median age at initiation of the second biologic was 12.2 years (range, 10.1–15.6). The median duration of treatment with the first biologic was 14.5 months (IQR, 11–26.25).

The most common comorbidities were allergic rhinitis, reported in 75% of patients, and atopic dermatitis, observed in 50%. Food allergy was present in one patient (12.5%). Other comorbidities included prematurity (*n* = 2), attention-deficit/hyperactivity disorder (ADHD) (*n* = 1), and Prader–Willi syndrome (*n* = 1).

All patients (100%) were sensitized to at least one perennial aeroallergen. At initiation of the first biologic, the median total serum IgE level was 1304 kU/L (IQR, 945–1776), the median blood eosinophil count was 590 cells/mm^3^ (IQR, 422–710), and the median FeNO level was 59 ppb (IQR, 54–65).

Before initiation of the first biologic, all patients underwent a comprehensive diagnostic evaluation for severe asthma, including chest computed tomography and bronchoscopy, to exclude alternative or coexisting causes of persistent respiratory symptoms. Further investigations for conditions such as vocal cord dysfunction, tracheobronchomalacia, immunodeficiency, primary ciliary dyskinesia, and cystic fibrosis were performed as clinically indicated.

### 3.2. Reasons for Biologic Switch

**Figure 1:**
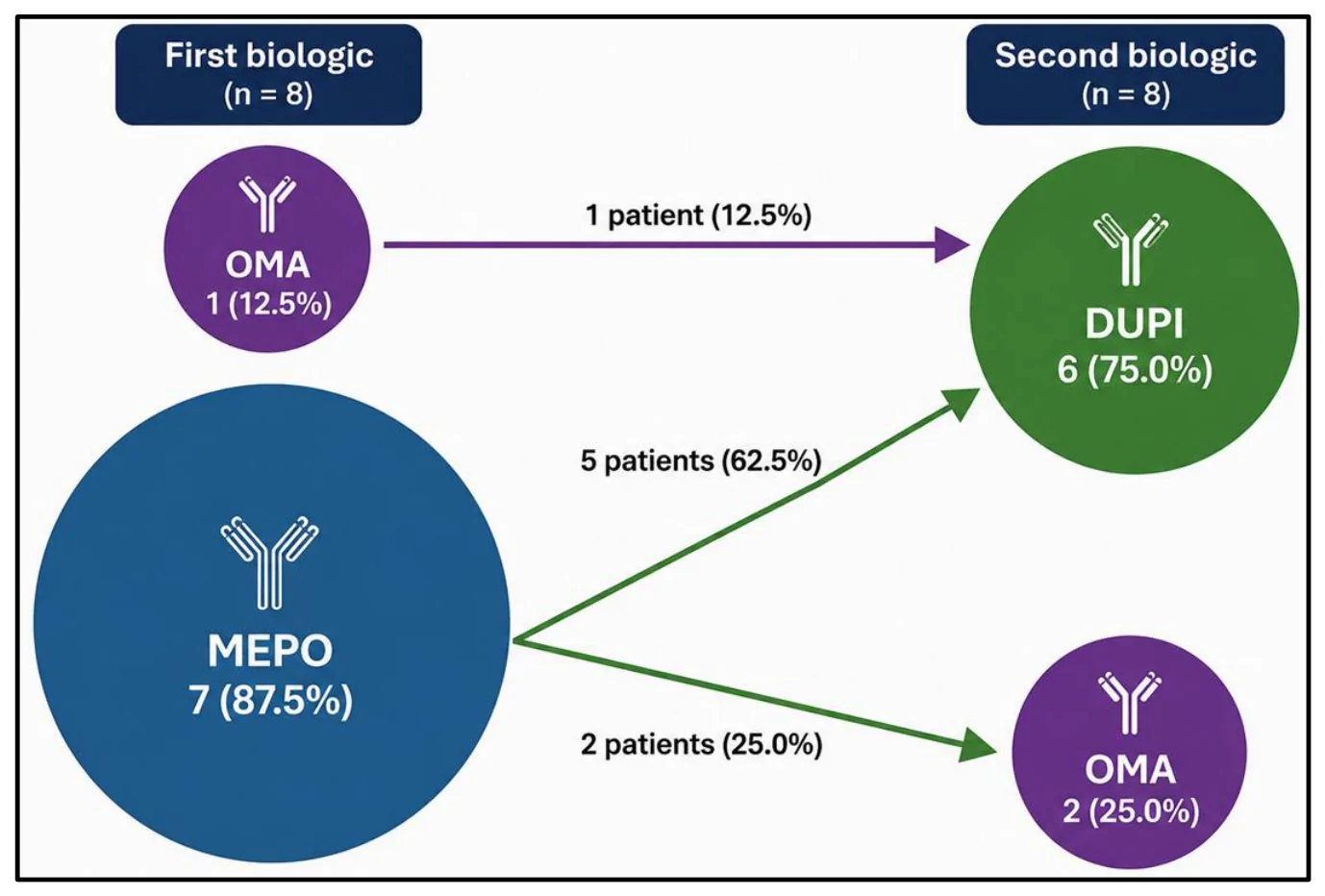
Patterns of biologic treatment switching. The figure illustrates transitions from the first to the second biologic therapy. OMA, omalizumab; MEPO, mepolizumab; DUPI, dupilumab.

As shown in Figure 1, omalizumab was the initial biologic in one patient (12.5%), whereas mepolizumab was used as first-line biologic therapy in the remaining seven patients (87.5%). Following treatment switch, the second biologic was omalizumab in two patients (25.0%) and dupilumab in the remaining six patients (75.0%).

Before establishing the lack of response and considering a biologic switch, adherence to inhaled corticosteroid-containing therapy and biologic injections, inhaler technique, comorbidities—including rhinosinusitis, allergic rhinitis, atopic dermatitis, and psychosocial factors—and relevant environmental exposures, including second-hand smoke and allergens, were systematically reassessed and excluded.

Qualitative analysis of the reasons for the switch of treatment showed that the primary indication for the switch of biologic therapy was inadequate asthma control in seven out of eight patients (87.5%), while one patient (12.5%) underwent treatment switch because of inadequate control of atopic dermatitis. The patient with atopic dermatitis showed an EASI score of 38 and a SCORAD score of 63 which decreased to 8 and 27 respectively after 4 months of treatment. Among patients with uncontrolled asthma, three (37.5% of the total cohort) experienced a high frequency of exacerbations, three (37.5%) had persistent moderate-to-severe airflow obstruction with reduced lung function, and one patient (12.5%) presented with both frequent exacerbations and persistently impaired lung function. None of the switches was due to side effects related to the first biologic; likewise, no adverse events related to the second biologic were recorded.

### 3.3. Pulmonary Function

**Figure 2:**
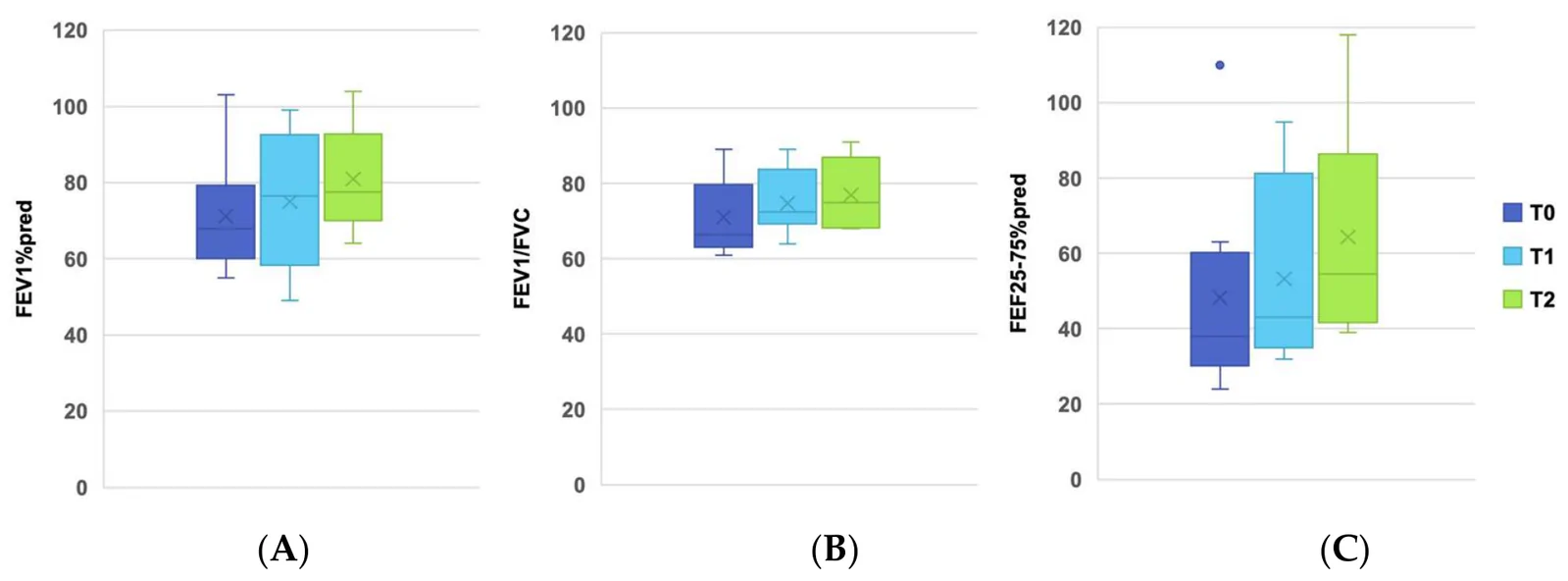
Changes in pulmonary function parameters after biologic switch. (**A**) FEV1 (% predicted). (**B**) FEV1/FVC ratio. (**C**) FEF25–75 (% predicted). Measurements were obtained before biologic switch (T0) and after 4 months (T1) and 12 months (T2). Data are presented as box-and-whisker plots, with the mean indicated by a cross, the median by a horizontal line, the boxes representing the interquartile range (IQR), and the whiskers indicating the minimum and maximum non-outlier values. Repeated-measures ANOVA demonstrated significant improvements in FEV1 (*p* = 0.002) and FEV1/FVC (*p* = 0.015), whereas the increase in FEF25–75 did not reach statistical significance (*p* = 0.08).

As shown in Figure 2, patients exhibited bronchial airflow obstruction at the time of biologic switch (T0), with a media FEV1 of 71.1% predicted (SD 15.4) and a media z-score of −2.33 (SD 1.3). FEV1 progressively increased at both follow-up visits (T1 after 4 months and T2 after 12 months). At T1, the media FEV1 was 75.0% predicted (SD 17.9), corresponding to a media z-score of −2.0 (SD 1.5). At T2, the media FEV1 further increased to 81.0% predicted (SD 14.2), with a media z-score of −1.5 (SD 1.1). Comparison of FEV1 values demonstrated a statistically significant improvement from baseline at both 4 and 12 months after the biologic switch (*p* = 0.002).

As shown in Figure 2, the media FEV1/FVC ratio at T0 was 71% (SD 10.3). Consistent with the improvement in FEV1, the FEV1/FVC ratio progressively increased during follow-up, reaching a media of 74.8% (SD 8.8) at T1 and 77% (SD 9.6) at T2. Comparison of FEV1/FVC values demonstrated a statistically significant improvement between baseline and both follow-up assessments (*p* = 0.015).

As shown in Figure 1, the media FEF25–75 at T0 was 48.3% predicted (SD 28.0), with a media z-score of −2.6 (SD 1.3). FEF25–75 showed a gradual increase over time, although the overall change did not reach statistical significance (*p* = 0.08). In detail, at T1, the media FEF25–75 was 53.3% predicted (SD 25.4), corresponding to a media z-score of −2.3 (SD 1.4), at T2, the media FEF25–75 was 64.4% predicted (SD 28), with a media z-score of −1.7 (SD 1.3).

### 3.4. Asthma Control

The median number of asthma exacerbations during the 12 months preceding biologic switch was 3 (IQR, 2.8–4). During the first 12 months of treatment with the second biologic, the median number of exacerbations decreased to 0 (IQR, 0–0.3). Comparison between baseline (T0) and 12-month follow-up (T2) demonstrated a statistically significant reduction in exacerbations requiring treatment with oral corticosteroids (*p* = 0.03). Moreover, at T0 the median fluticasone-equivalent ICS dose was 500 μg/day (IQR, 500–500), while twelve months after initiation of the second biologic, the median dose decreased to 425 μg/day (IQR, 200–500), although this reduction was not statistically significant (*p* = 0.125).

At baseline (T0), one patient (12.5%) had uncontrolled asthma according to GINA score, four (50.0%) had partially controlled asthma (GINA score 1 or 2), and three (37.5%) had controlled asthma (GINA score 0). At T1 (4 months), no patients had uncontrolled asthma (GINA score 3 or 4), while two (25.0%) had partially controlled asthma and six (75.0%) had controlled asthma. At T2 (12 months), no patients had uncontrolled asthma, one (12.5%) had partially controlled asthma, and seven (87.5%) had controlled asthma. Overall, GINA asthma control scores improved from T0 to T2; however, the change did not reach statistical significance (Friedman test, *p* = 0.09).

### 3.5. FeNO

Among Th2-markers, we analyzed FeNO level: at the moment of the switch it was 67 ppb (IQR, 67–88), while at 12 months after initiation of the second biologic (T2) the median FeNO level decreased to 13 ppb (IQR, 11–14.5).

### 3.6. Quality of Life

PAQLQ data at 12 months after initiation of the second biologic (T2) were available for only five out of the eight patients. The median total PAQLQ score at T2 was 6.4 (IQR, 5.9–6.7), with a possible maximum score of 7, indicating a good overall health-related quality of life. Domain-specific median scores were 6.1 (IQR, 5.9–6.2) for symptoms, 6.4 (IQR, 6.0–7.0) for activity limitation, and 6.9 (IQR, 6.4–6.9) for emotional function.

## 4. Discussion

In the present study, we retrospectively analyzed the clinical and functional outcomes of pediatric patients with severe asthma followed at our Pediatric Allergy and Respiratory Medicine Unit who required switching to a different biologic therapy. Inadequate asthma control was the primary indication for biologic switching. Switching to a second biologic was associated with sustained improvements in clinical outcomes and lung function, reduced maintenance inhaled corticosteroid requirements, and satisfactory health-related quality of life after 12 months of follow-up.

Among patients who underwent biologic switching, seven out of eight had initially been treated with mepolizumab. Although our data are referred to a small population and they are not generalizable, they might be consistent with previous reports showing that, although mepolizumab demonstrates substantial efficacy in adults with severe eosinophilic asthma, its clinical benefit appears to be less pronounced in the pediatric population [5,9].

It should also be acknowledged that regulatory prescribing criteria may have influenced the choice of the initial biologic therapy. At the time these patients initiated biologic treatment, dupilumab was approved in Italy only for patients aged ≥ 12 years, whereas mepolizumab was available for younger children. By the time biologic switching was considered, dupilumab had also received approval for children aged ≥ 6 years, thereby expanding the available therapeutic options in those 6 to 11 years old.

The median duration of treatment with the first biologic was 14.5 months, ranging from 8 to 38 months. The considerable variability in treatment duration likely reflects the lack of clear evidence-based recommendations regarding the minimum duration of biologic therapy and the criteria to be fulfilled before switching to an alternative agent [12].

In our cohort, the primary reason for switching biologic therapy was inadequate asthma control. Three of the eight patients (37.5%) experienced a high exacerbation burden, defined as more than three exacerbations during the 12 months preceding the switch, while three more patients (37.5%) had persistent symptoms associated with suboptimal lung function and moderate-to-severe persistent airflow obstruction. One patient presented with both frequent exacerbations and impaired lung function. We used no pre-specified cut-offs to establish the lack of response, but we applied a multidimensional approach, considering exacerbation burden, symptom control and lung function. From a practical perspective, regular clinical reassessment is essential to identify suboptimal responders promptly and determine whether switching to a biologic targeting an alternative inflammatory pathway may offer greater clinical benefit. For this reason, we applied an individualized switching strategy based on the patient’s evolving clinical profile rather than on a single outcome measure [13,14].

At the time of biologic switching, most patients exhibited impaired lung function. Specifically, five of the eight patients (62.5%) had moderate-to-severe airflow obstruction, one patient (12.5%) had mild airflow obstruction, and the remaining two patients (25.0%) had spirometric values at the lower limits of normal. Following the switch to a second biologic, lung function improved as early as the 4-month follow-up, with further improvement observed at the 12-month assessment. Given that most patients in our cohort were switched to dupilumab, these findings are consistent with the available evidence. Clinical studies evaluating dupilumab have demonstrated significant improvements in FEV_1_ as early as 2 weeks after treatment initiation, accompanied by parallel improvements in the FEV_1_/FVC ratio [15,16,17,18,19].

Interestingly, in our cohort at the 4-month follow-up none of the children showed uncontrolled asthma. This finding, combined with lung function data, suggests that a meaningful insight into treatment efficacy can already be obtained after four months.

Given that frequent exacerbations represented one of the main indications for biologic switching in 4 of the 8 patients, the observed reduction in exacerbation frequency suggests that switching biologic therapy was effective in improving this clinically relevant outcome [20]. The annual exacerbation rate significantly decreased 12 months after initiation of the second biologic (*p* = 0.03). These findings are in agreement with the available literature, which has consistently shown that treatment with omalizumab and dupilumab significantly reduces asthma exacerbations and the need for oral corticosteroids [17,19,21,22]. Moreover, our findings support the role of biologic switching as a promising therapeutic strategy for achieving clinical remission [23].

Within 12 months of initiating the second biologic, the daily ICS dose could be reduced from high- to medium-dose fluticasone equivalents in four of the eight patients. Although this reduction did not reach statistical significance, it represents a clinically meaningful benefit, as it may reduce the risk of ICS-related adverse effects, even though these are generally mild. Moreover, reducing the overall steroid burden is considered one of the key therapeutic goals of biologic therapy in severe asthma.

Finally, in our study, FeNO was monitored as non-invasive biomarker of T2 inflammation. At 12 months after initiation of the second biologic, FeNO levels had markedly decreased, with all patients achieving values below 20 ppb. This reduction may indicate improved suppression of type 2 airway inflammation after switching and, in patients treated with dupilumab, is biologically consistent with the inhibition of IL-4/IL-13 signaling, particularly the IL-13 pathway involved in nitric oxide production [24,25,26]. Our findings are in line with previous reports showing a marked reduction in FeNO during dupilumab therapy [24,25,26]. Nevertheless, FeNO may be influenced by corticosteroid exposure, treatment adherence, allergen exposure, and other clinical factors. Therefore, given the small sample size and absence of comprehensive longitudinal biomarker data, the observed reduction cannot be attributed exclusively to the second biologic and should be interpreted cautiously.

The inadequate response to the first biologic and the subsequent clinical improvement observed after switching may reflect an underlying pathophysiological pattern. In children, type 2 inflammation may evolve over time because of immunological maturation, while biomarkers may fluctuate according to disease activity, treatment exposure, environmental factors, and comorbidities [10,26]. Consequently, the inflammatory endotype identified when the first biologic was initiated may not have remained stable or may not have adequately reflected the dominant pathway driving the disease, resulting in a potential mismatch between the patient’s endotype and the selected biologic target. The favorable response to the second biologic could therefore be related to more appropriate targeting of the prevailing inflammatory pathway. However, direct evidence supporting this hypothesis is currently lacking, and the small, retrospective nature of our cohort does not allow this possibility to be confirmed or excluded.

The main limitation of this study is the small sample size, which reflects the rarity of the clinical scenario investigated. Indeed, biologic switching is required in only a minority of children with severe asthma, a condition that is itself uncommon. In addition, the retrospective design limited our analyses to the clinical and functional data routinely collected during patients’ follow-up, preventing a more comprehensive assessment of potential predictors of treatment response. Furthermore PAQLQ data were available only at T2 and not at baseline. Consequently, changes in health-related quality of life over time could not be assessed, limiting our ability to evaluate the impact of biologic switching on this relevant patient-reported outcome.

Finally, inflammatory biomarkers were not comprehensively assessed. Although FeNO measurements were consistently available, total IgE levels and blood eosinophil counts were not systematically measured longitudinally. Consequently, neither the levels nor the changes over time in these biomarkers contributed to the decision to switch biologic therapy in clinical practice at our center.

A major strength of this study is that it reflects real-world clinical experience and decision-making at a tertiary referral center, providing valuable insights into the management of pediatric severe asthma. Nonetheless, larger prospective studies are needed to establish standardized criteria and the optimal timing for biologic switching in pediatric patients with severe asthma. Future studies should also incorporate a longitudinal evaluation of patient-reported outcome measures, including the Asthma Control Test (ACT) and the Pediatric Asthma Quality of Life Questionnaire (PAQLQ), which in our study was evaluated only at T2.

## 5. Conclusions

In conclusion, in our cohort, the primary indication for biologic switching was inadequate asthma control despite ongoing biologic therapy. Switching to a second biologic was associated with significant clinical and functional improvement, which was already evident at the 4-month follow-up and was sustained after 12 months of treatment. Although a longer follow-up may be desirable and we observed our patients for at least six months before switching, these findings suggest that, after only four months, a meaningful insight into treatment efficacy can be obtained through a multidimensional assessment. Nonetheless, before concluding that a biologic has failed, adherence to therapies, alternative or coexisting diagnoses, comorbidity control, psychosocial factors, and relevant environmental exposures should be systematically reassessed and addressed. Moreover, in children, biologic selection should also be periodically reconsidered as the patient grows older and becomes eligible for additional age-licensed agents, possibly more suited to the patient’s phenotype or comorbidities. Taken together, our findings support a frequent multidimensional reassessment of children, especially during the first months of treatment with a biologic therapy. This individualized approach may facilitate the early identification of suboptimal responders and guide timely switching to an alternative biologic.

## Figures and Tables

**Table 1:** Summary at the patient-level of the biologic treatment sequences, baseline blood eosinophils and total serum IgE, and reasons for switching. Pt = patient.

	Sex	Baseline Blood Eosinophils (/μL) and Total Serum IgE (KU/L)	First Biologic	Reason for Discontinuation of the first Biologic	Second Biologic
Pt 1	M	170/μL and 682 KU/L	Omalizumab	Poor asthma control (high frequency of exacerbations) and reduced lung function	Dupilumab
Pt 2	F	1250/μL and 1033 KU/L	Mepolizumab	Poor asthma control	Dupilumab
Pt 3	M	830/μL and 1803 KU/L	Mepolizumab	Poor asthma control and reduced lung function	Dupilumab
Pt 4	F	430/μL and 1767 KU/L	Mepolizumab	Poor asthma control and reduced lung function	Dupilumab
Pt 5	F	570/μL and 1816 KU/L	Mepolizumab	Poor atopic dermatitis control	Dupilumab
Pt 6	M	400/μL and 254 KU/L	Mepolizumab	Poor asthma control (high frequency of exacerbations)	Omalizumab
Pt 7	M	670/μL and 1300 KU/L	Mepolizumab	Poor asthma control (high frequency of exacerbations)	Omalizumab
Pt 8	M	610/μL and 1308 KU/L	Mepolizumab	Poor asthma control and reduced lung function	Dupilumab

## Data Availability

Data is unavailable due to privacy or ethical restrictions.
